# Physical activity of Estonian family doctors and their counselling for a healthy lifestyle: a cross-sectional study

**DOI:** 10.1186/1471-2296-11-48

**Published:** 2010-06-18

**Authors:** Kadri Suija, Ülle Pechter, Jaak Maaroos, Ruth Kalda, Anneli Rätsep, Marje Oona, Heidi-Ingrid Maaroos

**Affiliations:** 1 Department of Polyclinic and Family Medicine, University of Tartu, Tartu, Estonia; 2 The Clinic of Sports Medicine and Rehabilitation, Tartu University Hospital, Tartu, Estonia

## Abstract

**Background:**

Physical activity offers major health benefits and counselling for it should be integrated into the medical consultation. Based on the literature, the personal health behaviour of the physician (including physical activity) is associated with his/her approach to counselling patients. Our hypothesis is that family doctors (FD) in Estonia are physically active and their recommendation to counsel patients with chronic diseases to use physical activity is high. The study was also interested in how FDs value physical activity among other important determinants of a healthy lifestyle, e.g. nutrition, non-consumption of alcohol, and non-smoking.

**Methods:**

Physicians on the electronic list were contacted by e-mail and sent a questionnaire. The first part assessed physical activity by the International Physical Activity Questionnaire (IPAQ) short form. Self-reported physical activity during one week was calculated as total physical activity in minutes per week (MET min/week). The second part of the questionnaire included questions about the counselling of patients with chronic disease concerning their physical activity and a healthy lifestyle. The study focused on female FDs because 95% of the FDs in Estonia are women and to avoid bias related to gender.

**Results:**

198 female FDs completed the questionnaire. 92% reported that they exercised over the past 7 days to a moderate or high level of physical activity. Analysis revealed no statistically significant relationship between the level of physical activity and general characteristics (age, living area, body mass index [BMI], time spent sitting). FDs reported that patients with heart problems, diabetes, and obesity seek their advice on physical activity more often than patients with depression. Over 94% of the FDs claimed that they counsel their patients with chronic diseases about exercising. According to the FDs' reports, the most important topic in counselling patients for a healthy lifestyle was physical activity.

**Conclusion:**

This study showed that female FDs are physically active. The level of physical activity is not related to their age, BMI, living area, or time spent sitting. Also, FDs reported that promotion of physical activity is part of their everyday work.

## Background

A number of recent studies have shown that regular physical activity is beneficial for patients with different health problems, e.g. cardiovascular, musculoskeletal, obesity, and emotional disorders [1-4]. As well as physical activity, the care of patients with chronic diseases must also address other factors concerning a healthy lifestyle, e.g. diet, non-smoking, and non-consumption of alcohol. Promotion of physical activity and counselling about a healthy lifestyle among patients is one of the physician's tasks. Family doctors (FD) are particularly well placed for health promotion: early enquiry about patients' lifestyles, provision of information, and counselling concerning risk factors [5]. Giving advice and educating patients about health-related risk factors are considered professional responsibility by physicians themselves and also expected by patients [6]. According to the literature, primary care doctors are more active in health promotion than other professionals [7,8] and women physicians are more likely to counsel regarding prevention than men [7]. Rogers et al [9] reported that the physician's personal experience of his/her own physical activity also improved counselling for physical activity. Doctors who exercised regularly were also more effective in helping patients to practise regular physical exercise [10,11]. Previous studies concerning the physical activity of physicians have given controversial results. However, with very few exceptions [12] most studies have shown that physicians are physically more active [11,13,14] compared to the general population. Physical activity seems to be associated with gender [2,13]. No studies have assessed physical activity of physicians in Estonia.

Our hypothesis is that FDs in Estonia are physically active and therefore their drive to counsel patients with chronic diseases to use physical activity is also high. We wished to know how FDs rate physical activity compared to other important determinants of a healthy lifestyle, e.g. nutrition, non-consumption of alcohol, and non-smoking.

## Methods

### Study group

From May 2009 to October 2009, we contacted by e-mail the physicians working in Estonia whose daily work involves consulting patients with different health problems and who subscribed to the electronic list of physicians. We asked them to participate in our study concerning the assessment of and counselling for physical activity. The questionnaire was largely filled in electronically using an electronic formular, the eFormular [15]. The eFormular is a unique web-based tool giving the possibility of creating electronic forms, conducting surveys, and collecting necessary information via the Internet [15]. It was also possible to complete a paper version of the questionnaire at the doctors' meeting.

**Figure 1:**
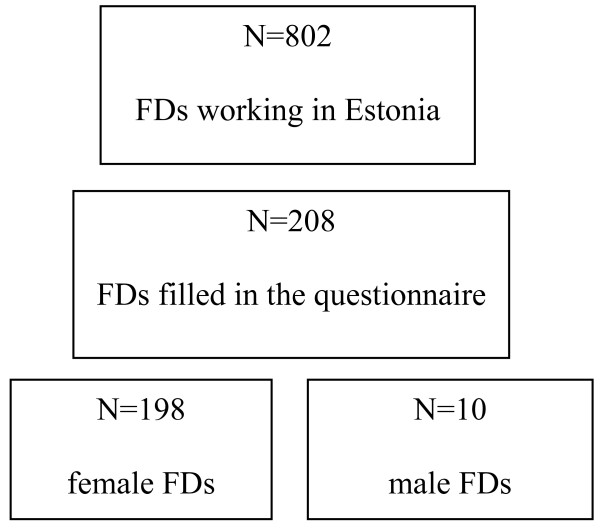
**Flowchart of the study population**.

Figure 1 presents the flowchart of the study population.

There are 802 FDs working in Estonia and 95% of them are women [16]. Of the 208 physicians who filled in the questionnaire, 177 used the electronic version and 31 the paper version. 95% of the FDs (N = 198) were women. Thus, female FDs formed the majority of the study group. Also there was a difference in the level of physical activity of the female and male FD's (mean MET of the men 3743 vs. 2871 of the women). Therefore, we excluded the few men from the sample and only report on the women.

### Instruments/measurement

The first part of the questionnaire assessed physical activity. The International Physical Activity Questionnaire (IPAQ) short form was chosen to assess physical activity because of its high reliability and validity [17]. Although the IPAQ has been translated into many languages worldwide, there is no version in the Estonian language. In the translation process, relevant guidelines and recommendations were taken into account [17,18]. The procedure comprised forward translation, assessment of item comprehension, back-translation into English, and development of a consensual version based on the results of the previous translations and comprehension assessments. Two native Estonian speakers with an excellent knowledge of English completed an independent forward translation of the original IPAQ. The 2 translations were then reconciled, followed by back-translation into English by a third independent translator who had no access to the original version of the IPAQ. The back-translation and the original versions of the questionnaire were compared of performed and the results were used for the development of the final consensual translation of the Estonian-language IPAQ.

The IPAQ short version estimates how much health enhancing physical activity, including daily life activities and exercise, the person has undertaken over the previous 7 days.

Physical activity is defined as any bodily movement produced by skeletal muscles which results in energy expenditure that can be categorized into occupational, sports, conditioning, household, or other active daily life activities [19].

Daily life activities are known as the tasks of everyday life that are normal and independently performed, including self-care activities, such as eating, walking, bathing, and dressing, or more complicated activities, such as working, homemaking, and shopping [20].

Exercise is a specific type of physical activity that is planned, structured, and repeatedly done to improve or maintain physical fitness [19].

The IPAQ included questions about physical activity of 3 intensities (vigorous physical activity, moderate physical activity, and walking). The individual had to estimate how many days (frequency) he/she was physically active and the average time (duration) that he/she spent being physically active on these days. We calculated the total physical activity, MET or metabolic equivalent (MET min/week), as suggested in the Guidelines for Data Processing and Analysis of the International Physical Activity Questionnaire for the sum of walking, and moderate, and vigorous physical activity [18]. The selected MET values were derived from the IPAQ Reliability Study [17]. Using the compendium of Ainsworth et al [21], an average MET score was derived for each type of activity.

The second part of the questionnaire assessed counselling. This included 3 questions: whether patients with different health problems (coronary heart disease, hypertension, type-2 diabetes, depression, and obesity) seek a physician's advice on physical activity; whether family doctors counsel patients with chronic diseases (coronary heart disease, hypertension, diabetes 2, depression, obesity) about physical activity; and their opinion about the importance of physical activity among some other determinants of healthy lifestyle (non-smoking, non-consumption of alcohol, healthy nutrition) according to the contribution to health in the process of counselling patients with chronic disease. Later data about coronary heart disease and hypertension were integrated as heart disease.

These new questions were added and based on evidence of the effectiveness of physical activity on the management of chronic health problems [4,22], and also based on the importance of counselling of physical activity by physicians [5,23].

The third part of the questionnaire included questions about the participants' age, height and weight, gender, place of residence (rural or urban), and speciality.

Body mass index (BMI) was calculated using height and weight.

The questionnaire was anonymous and voluntary and took about 10 minutes to complete [additional file 1]. It was first tested on a pilot study group of 25 participants (all FDs) for comprehensibility of the questionnaire; no problems were reported.

### Statistics

The Statistical Package for the Social Sciences (SPSS) for Windows Release 17.0.0 was used for data analysis [24]. Standard methods: the mean, standard deviation (SD), median, and % were employed for descriptive statistics. Differences between the physicians with low, moderate, and high physical activity were analysed with the Chi-square test. All tests were 2-sided and statistical significance was taken as p < 0.05.

### Ethics

The Ethics Committee of the University of Tartu approved the study.

## Results

### Physical activity among physicians

Table 1 presents general characteristics of the study group.

**Table 1:** General characteristics of the study group

Characteristic	Number (n)	%
Total	198	100

**Age**		
≤39	41	21
40-59	140	71
≥60	17	8

**Place of residence**		
Urban	133	67
Rural	65	33

**BMI**		
< 25	125	63
25-29.9	56	28
≥30	17	9

**Physical activity (MET min/week)**		
Low ≤600	16	8
Moderate 601-3000	117	59
High ≥3001	65	33

**Sitting in minutes/day**		
≤360	88	44
> 360	110	56

It consisted of 198 female FDs. About one third of them (33%) lived in a rural area. The age-distribution of the physicians given in Table 1 shows that the mean age of the physicians was 47.1 ± 9.4 years (median 47.0). Normal (BMI < 25) was reported by 63% of the physicians (Table 1). The mean BMI was 24.6 ± 3.6 (median 24.0). 59% of the physicians reported having exercised using a moderate physical activity (MET 601-3000) and 34% reported having exercised using a high physical activity (MET≥3001) during the past 7 days. The mean physical activity of the physicians was 2871.0 ± 2470.0 MET min/week (median 2106.0). The mean time (minutes/day) spent on sitting was 396.6 ± 139.9 (median 400.0). Sitting more than six hours per day was reported by 56% of the physicians (Table 1).

The general characteristics of the physicians according to their physical activity (Table 2) shows no significant difference in the characteristics analysed (age in groups, place of residence, BMI, sitting min/day) between the physicians with low, moderate, and high physical activity.

**Table 2:** General characteristics of the FDs according to their level of physical activity

Characteristic	Low physical activity n (%)	Moderate physical activity n (%)	High physical activity n (%)	p-value
**Age**				0.355
≤39	2 (5)	27 (66)	12 (29)	
40-59	10 (7)	81 (58)	49 (35)	
≥60	4 (23)	9 (53)	4 (23)	

**Place of residence**				0.316
Urban	13 (10)	78 (58)	42 (32)	
Rural	3 (5)	39 (60)	23 (35)	

**BMI**	9 (7)	70 (56)	46 (37)	0.131
< 25	7 (10)	47 (64)	19 (26)	
≥25				

**Sitting min/day**				0.207
≤360	5 (6)	51 (58)	32 (36)	
> 360	11 (10)	66 (60)	33 (30)	

### Counselling patients for physical activity

**Figure 2:**
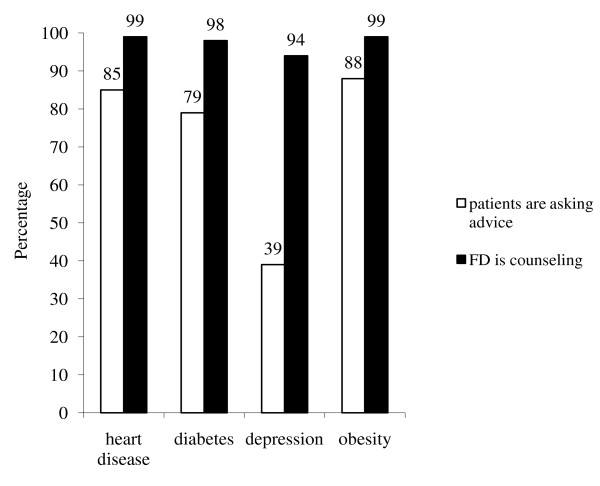
**How often patients with certain chronic health problems seek advice from their FD and how often the FDs counsel patients for physical activity according to the FDs self-report**.

Figure 2 shows the answers to questions of how often patients with certain chronic disease (heart disease, diabetes 2, depression, obesity) seek advice from their FD and how often the FDs counselled patients to use physical activity. According to the FDs self-reports, patients with heart problems, diabetes, and obesity seek their advice on physical activity more often than patients with depression (Figure 2).

FDs claimed that they counsel over 94% of their patients about physical activity (Figure 2).

### Determinants of healthy lifestyle

Of the most important factor contributing to health in the process of counselling patients with chronic disease, 43% of the FDs said it was physical activity, 42% non-smoking, 39% healthy food or nutrition, and 24% non-consumption of alcohol.

## Discussion

Female FDs claim to be physically active, a low level of physical activity being reported by < 10% of them. According to the Estonian Health Interview Survey 2006 involving 6434 Estonian residents aged 15-84 years, ~70% of the respondents had undertaken moderate physical activity during the past 4 weeks [25]. Our result that Estonian FDs are physically more active compared to the general population, is in line with most of the previously conducted studies [11,13,14]. The postal survey among FDs from 11 European countries, including Estonia, found that 35% of the FDs from Estonia exercised regularly [10]. In comparison, 43% of general practitioners were physically inactive in the PHIT GP Survey among general practitioners in Ireland that used IPAQ [14]. Heterogeneity in assessment tools makes difficult to compare the exercise habits of physicians. However, our result that physicians are physically active is comparable to the results of other studies [10,11,13,14].

The physician's level of physical activity (low, moderate, or high) was not related to the analysed contributing factors. Thus, we cannot claim that physical activity depends on the age, place of residence, BMI, or time spent on sitting by the physician.

FDs who were studied were physically active and promoted physical activity among patients through counselling. 94% of our population claimed that they counselled their patients about exercise. Earlier studies have reported lower levels of counselling of patients [5,7,10]. Since the percentage of physicians counselling patients was so high, we were not able to differentiate high vs. low counsellors. Our high level of counselling could be influenced by our sample; women physicians are more likely to counsel regarding prevention than men [7], and primary care doctors are more active in health promotion than other professionals [7,8].

We found that patients are also active in asking advice about physical activity. Patients with heart problems, diabetes, and obesity seek medical advice on physical activity more often than patients with depression. This could be related to the poor motivation of depressed patients to take exercise [26]. Based on our earlier study, both the level of previous physical activity of depressed patients and their motivation to exercise regularly were low [27]. Even when patients' reluctance to start regular physical activity was high, exercising and using physical activity was shown to improve their mood [27]. Obviously, FDs should pay more attention to the lifestyle of depressed patients since such patients appear too passive to seek independently advice from FDs.

There are other factors besides physical activity that determine a healthy lifestyle. We found that the most important factor contributing to health in the process of counselling patients with chronic disease was physical activity followed by non-smoking, healthy nutrition, and non-consumption of alcohol. According to a study conducted among primary care physicians in the UK, the most important issue in promoting good health in patients was non-smoking followed by regular exercising [5]. However, for a quarter of the physicians (26%) in the present study, reporting the most important factor contributing to health in counselling for a healthy lifestyle proved difficult. To some extent this is understandable because none of the factors - healthy nutrition, physical activity, non-smoking, or non-consumption of alcohol - alone is definitely the most significant in any given situation. As counselling depends on the patient, his/her risks, health problems, and lifestyle, it is essential to be flexible and patient-centred. Thus physical activity should always be prescribed on an individual basis [28]. According to Ampt et al [6], the amount of information given by the physician should be proportional to patient risk. Hence, it is more important to discuss a healthy lifestyle and motivate patients according to his/her problems. Physicians should focus more on how to integrate physical activity into regular daily activities, for example, walking.

In modifying lifestyle or habits, it is essential that both partners communicate actively in order to share information with one another and co-operate to help solve the problem [29]. The patient's level of motivation is possibly one of the most important factors influencing counselling and changing lifestyle. The physician's knowledge can also influence counselling [5,6]. A physician's behaviour is affected by his/her general attitude to the importance of preventive care [6], and those who regard exercise as a highly important health contributing factor are more likely to counsel for exercise [11]. Consequently, one of our aims was to bring physical activity into focus among physicians by using the questionnaire as a tool.

The strength of the study is the use of the IPAQ, which is designed to assess self-reported physical activity and standardize measurements of physical activity in different independent studies. Thus our results are comparable to findings from similar physical activity studies based on the above widely used questionnaire.

The study also has some limitations, one being limitation is the sample. Although, the sample was quite small and the response rate was low, it involved ~25% of the FDs working in Estonia. It consisted only of female FDs, as 95% of the FDs in Estonia are women [16]. According to the literature, physical activity is influenced by gender [2]. We also found that the level of physical activity of the men was higher than of the women (mean MET of the men 3743 vs. 2871 of the women). Therefore, we excluded the few men from the sample and only reported on the women. Also the mean age of the study group is comparable to the mean age of Estonian FDs [16]. Thus, we think the sample is representative of family doctors in Estonia.

Another limitation is the possible risk of overestimation or underestimation where physical activity is self-reported. The self-reported total physical activity scores alone do not yield a complete pattern of physical activity. On the other hand, the questionnaire is the most widely used method in epidemiological studies, while laboratory methods are more expensive and mainly employed for validation purposes [30]. Hence it is evident that validated self-reported questionnaires like the IPAQ are suitable for everyday practice [17].

## Conclusions

The most important finding of the study is that the women physicians reported being physically active. Another major finding is that promotion of health and especially the encouraging of physical activity among patients is part of the physicians' everyday work. According to the physicians' reports, patients with chronic health problems seek advice on physical activity. It appeared that only patients with depression were more passive in this respect. Therefore, more attention should be paid to the counselling of depressed patients.

## List of abbreviations

BMI: body mass index; FD: family doctor; IPAQ: International Physical Activity Questionnaire; SD: standard deviation; SPSS: Statistical Package for the Social Sciences.

## Competing interests

This study was funded by the Estonian Science Foundation (grant No. 7596) and by target financing (TARPO 0821).

The authors declare that they have no conflicting interests.

## Authors' contributions

KS participated in the designing of the study, collected and analysed the data, and completed the manuscript. ÜP participated in the designing of the study, collected and analysed the data, and participated in writing the manuscript. HIM and JM designed the study, participated in data analysis, and approved the final manuscript. RK, AR, and MO participated in the designing of the study and approved the final manuscript.

All authors read and approved the final manuscript.

## Pre-publication history

The pre-publication history for this paper can be accessed here:

http://www.biomedcentral.com/1471-2296/11/48/prepub

## Supplementary Material

Additional file 1 **Questionnaire**: - I International Physical Activity Questionnaire - II Counselling for physical activity in your practice - III Please include the following data about yourself. Click here for file
