# Supplementary material for: Physical activity of Estonian family doctors and their counselling for a healthy lifestyle: a cross-sectional study
Source: BMC Fam Pract. 2010 Jun 18;11:48. doi: 10.1186/1471-2296-11-48 (PMC2909965; doi:10.1186/1471-2296-11-48)
Supplement: Additional file 1 — Questionnaire: - I International Physical Activity Questionnaire - II Counselling for physical activity in your practice - III Please include the following data about yourself. [file 1471-2296-11-48-S1.PDF]

I INTERNATIONAL PHYSICAL ACTIVITY QUESTIONNAIRE

We are interested in finding out about the kinds of physical activities that people do as part of their everyday lives. The questions will ask you about the time you spent being physically active in the **last 7 days**. Please answer each question even if you do not consider yourself to be an active person. Pease think about the activities you do at work, as part of your house and yard work, to get from place to place, and in spare time for recreation, exercise or sport.

Think about all the vigorous activities that you did in the **last 7 days. Vigorous** physical activities refer to activities that take hard physical effort and make you breathe much harder than normal. Think *only* about those physical activities that you did for at least 10 minutes at a time.

1. During the **last 7 days,** on how many days did you do **vigorous** physical activities like heavy lifting, digging, chopping wood, speed skiing, running, aerobics, tennis or fast bicycling?

   _____ **days per week**

   ☐ No vigorous physical activities → ***Skip to question 3***

2. How much time did you usually spend doing vigorous physical activities on one of those days?

   _____ **hours per day**

   _____ **minutes per day**

   ☐ Don’t know / Not sure

Think about all the **moderate** activities that you did in the **last 7 days. Moderate** activities refer to activities that take moderate physical effort and make you breathe somewhat harder than normal. Think about those physical activities that you did for at least 10 minutes at a time.

3. During the **last 7 days,** on how many days did you do **moderate** physical activities like carrying light loads, bicycling at a regular pace, Nordic walking, roller skating or doubles tennis? Do not include walking.

   _____ **days per week**

   ☐ No moderate physical activities → ***Skip to question 5***

4. How much time did you usually spend on **moderate** physical activities on one of those days?

_____ **hours per day**

_____ **minutes per day**

☐ Don't know / Not sure

Think about the time you spent **walking** in the **last 7 days**. This includes at work and at home, walking to travel from place to place, and any other walking that you might do solely for recreation, sport, exercise, or leisure.

5. During the **last 7 days,** on how many days did you **walk** for at least 10 at a time?

_____ **days per week**

☐ No walking → ***Skip to question 7***

6. How much time did you usually spend **walking** on one of those days?

_____ **hours per day**

_____ **minutes per day**

☐ Don't know / Not sure

The last question is about the time you spent **sitting** on weekdays during the **last 7 days**. Include time spent at work, at home, while doing course work and during leisure time. This may include time spent sitting at a desk, visiting friends, reading, or sitting or lying down to watch television.

7. During the **last 7 day,** how much time did you spend **sitting** on a **week day**?

_____ **hours per day**

_____ **minutes per day**

☐ Don't know / Not sure

II COUNSELLING FOR PHYSICAL ACTIVITY IN YOUR PRACTICE

1. Whether patients with the chronic diseases listed below seek your advice on physical activity?

coronary heart disease	YES	NO
hypertension	YES	NO
type-2 diabetes	YES	NO
depression	YES	NO
obesity	YES	NO

2. Whether you counsel patients with the chronic diseases listed below about physical activity?

coronary heart disease	YES	NO
hypertension	YES	NO
type-2 diabetes	YES	NO
depression	YES	NO
obesity	YES	NO

3. Please rank the factors listed below, from the most important to the least important, according to your opinion about their contribution to health in the process of counselling of patients with chronic diseases.
1 – the most important 4 – the least important

Non-smoking	1	2	3	4
Non-consumption of alcohol	1	2	3	4
Healthy nutrition	1	2	3	4
Physical activity	1	2	3	4

III PLEASE INCLUDE THE FOLLOWING DATA ABOUT YOURSELF:

AGE____ WEIGHT____HEIGHT_____GENDER Female / Male

PLACE OF RESIDENCE RURAL / URBAN

SPECIALITY: Family doctor___
Other_________________________(please write)

Thank you for participation!
